# Fluoxetine increases plasticity and modulates the proteomic profile in the adult mouse visual cortex

**DOI:** 10.1038/srep12517

**Published:** 2015-07-24

**Authors:** L. Ruiz-Perera, M. Muniz, G. Vierci, N. Bornia, L. Baroncelli, A. Sale, F.M. Rossi

**Affiliations:** 1 Laboratorio de Neurociencias “Neuroplasticity Unit”, Facultad de Ciencias, UdelaR, Montevideo, Uruguay; 2 Institute of Neuroscience, Consiglio Nazionale delle Ricerche, Pisa, Italy

## Abstract

The scarce functional recovery of the adult CNS following injuries or diseases is largely due to its reduced potential for plasticity, the ability to reorganize neural connections as a function of experience. Recently, some new strategies restoring high levels of plasticity in the adult brain have been identified, especially in the paradigmatic model of the visual system. A chronic treatment with the anti-depressant fluoxetine reinstates plasticity in the adult rat primary visual cortex, inducing recovery of vision in amblyopic animals. The molecular mechanisms underlying this effect remain largely unknown. Here, we explored fluoxetine effects on mouse visual cortical plasticity, and exploited a proteomic approach to identify possible candidates mediating the outcome of the antidepressant treatment on adult cortical plasticity. We showed that fluoxetine restores ocular dominance plasticity in the adult mouse visual cortex, and identified 31 differentially expressed protein spots in fluoxetine-treated animals vs. controls. MALDITOF/TOF mass spectrometry identification followed by bioinformatics analysis revealed that these proteins are involved in the control of cytoskeleton organization, endocytosis, molecular transport, intracellular signaling, redox cellular state, metabolism and protein degradation. Altogether, these results indicate a complex effect of fluoxetine on neuronal signaling mechanisms potentially involved in restoring plasticity in the adult brain.

Critical periods for experience-dependent plasticity are specific developmental time windows during which the central nervous system displays a great potential for shaping neuronal circuits in response to sensory stimuli, a widespread process in the brain occurring from sensory systems (e.g. visual system) up to multimodal brain systems (e.g. human language). While a certain degree of plasticity is maintained in adulthood in some specific brain structures, allowing lifelong learning, the typical critical period heightened sensitivity to modifications induced by experience manipulations undergoes a dramatic decline with age in the sensory cortices^1^. Since this constitutes a major obstacle for potential functional recovery when the brain is damaged by traumas, pathologies or developmental defects, much effort is being currently done aimed at identifying new experimental strategies capable to modulate the mechanisms that control and limit neuronal plasticity in the adult.

Recent studies in the visual cortex of rodents have identified a few non invasive approaches for restoring juvenile-like levels of plasticity in the adult^2^,^3^. Among these, a long-term treatment with the very well known anti-depressant fluoxetine emerges as particularly interesting, given its high potential for a clinical application to humans^4^. It has been shown that fluoxetine reopens experience-dependent plasticity in the visual cortex of adult naïve rats (measured as sensitivity to monocular deprivation, MD) and leads to a full recovery of visual functions (visual acuity and binocularity) in adult animals rendered amblyopic by MD performed during the critical period^4^. These striking effects of fluoxetine have been associated to its ability to modulate brain levels of the neurotrophin BDNF, and the cortical excitatory/inhibitory balance, a major regulator of plasticity both during the critical period and in the adult^5^.

Despite subsequent attempts to characterize other molecular factors underlying the impact of fluoxetine on visual cortical plasticity in the rat^6^, no detailed information is currently available concerning the effects of fluoxetine on visual cortical plasticity in the mouse, a species suitable for genetic manipulation and thus highly interesting in terms of its experimental potential for further advancements in this field.

Here, we investigated the effects of fluoxetine on visual cortical plasticity in the adult mouse and explored, using a proteomic approach, possible proteins that may emerge as good molecular candidates underlying the impact of fluoxetine on neuronal plasticity in the adult visual cortex.

## Results

### Fluoxetine reactivates ocular dominance plasticity in the adult mouse visual cortex

**Figure 1:**
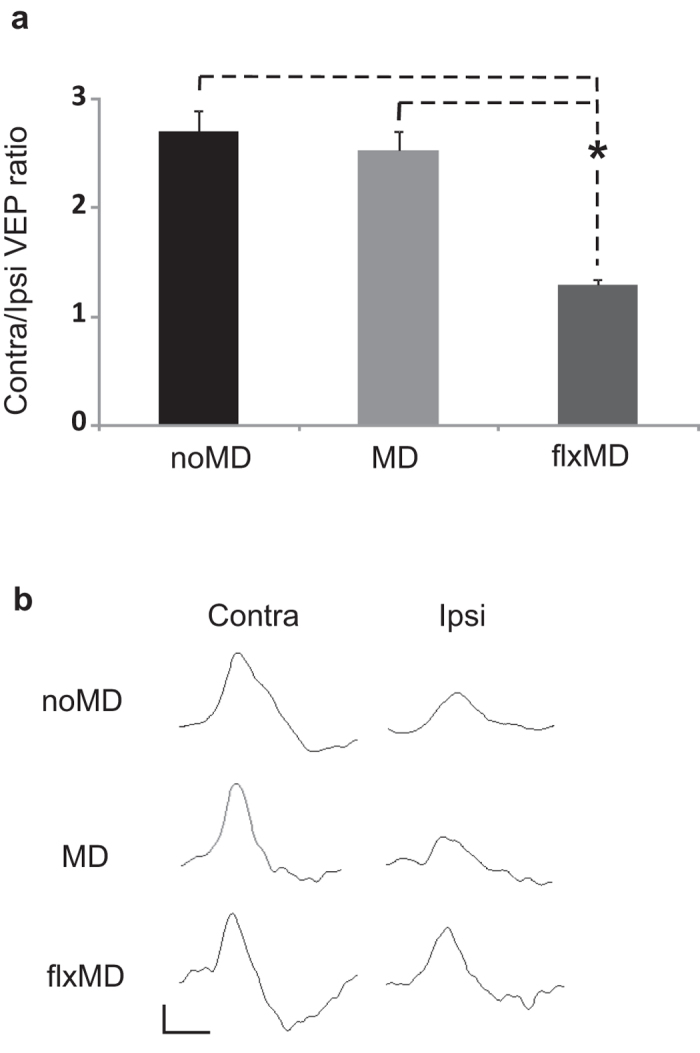
Fluoxetine reactivates ocular dominance plasticity in the adult mouse visual cortex. (**A**) Contralateral to ipsilateral eye (C/I) VEP ratio mean values in naive (noMD), monocularly deprived untreated (MD) and monocularly deprived fluoxetine-treated (flxMD) adult mice. The C/I VEP ratio is around 2.7 in naive adult animals (noMD), reflecting the predominance of crossed fibers in mouse retinal projections (noMD: n = 5, VEP ratio = 2.71 ± 0.19). VEP recordings revealed that 3 days of MD did not affect the C/I VEP ratio in monocularly deprived adult untreated mice (MD: n = 6, VEP ratio = 2.53 ± 0.18), whereas it led to a significant decrease in the C/I VEP ratio in fluoxetine-treated adult animals (flxMD: n = 6, VEP ratio = 1.30 ± 0.05). The statistical analysis showed a significant difference between the VEP ratio of flxMD mice and that of both noMD and MD animals (one-way ANOVA, post-hoc Holm–Sidak method, p < 0.05 in both cases); the VEP ratio of noMD and that of MD mice did not differ between each other (one-way ANOVA, post-hoc Holm–Sidak method, p = 0.417). (**B**) Examples of VEP responses to the stimulation of the contralateral or ipsilateral eye to the cortex in which the recording was performed in the three groups of animals. Calibration bars: 50 μV, 100 ms.

We investigated whether a chronic treatment with fluoxetine restores plasticity in the adult visual system of the mouse, using the classical model of ocular dominance (OD) shift of visual cortical neurons after three days of monocular deprivation (MD). This plastic phenomenon in the mouse is restricted to a critical period during postnatal development and is absent in the adult because of a decline of plasticity. We evaluated the effects of MD on OD plasticity of adult mice chronically treated with fluoxetine by recording visual evoked potentials (VEPs) in the binocular region of the primary visual cortex contralateral to the deprived eye. VEPs represent the integrated response of a population of neurons to patterned visual stimuli and are routinely used to evaluate visual acuity (VA) and binocularity alterations^4^,^7^. We assessed OD (binocularity) calculating the contralateral-to ipsilateral (C/I) VEP ratio; that is, the ratio of VEP amplitudes recorded by stimulating the eye contralateral and ipsilateral, respectively, to the visual cortex where recording is performed. As shown in Fig. 1, the C/I VEP ratio is around 2.7 in adult animals, reflecting the predominance of crossed fibers in mouse retinal projections (noMD: n = 5, VEP ratio = 2.71 ± 0.19). As expected, 3 days of MD did not affect the C/I VEP ratio in the visual cortex contralateral to the occluded eye in control untreated adult animals, confirming the absence of OD plasticity in adult mice in response to a brief period of MD (untreated MD mice, n = 6, C/I VEP ratio = 2.53 ± 0.18). In contrast, fluoxetine-treated adult mice showed a marked OD shift in favor of the non deprived eye after MD (flxMD mice: n = 6, C/I VEP ratio = 1.30 ± 0.05), thus displaying a plastic modification normally restricted to the early stages of brain development. The statistical analysis showed a significant difference between the C/I VEP ratio of flxMD mice and that of both noMD (naive animals) and untreated MD animals (one-way ANOVA, post-hoc Holm-Sidak method, p < 0.05 in both cases), whereas the C/I VEP ratio of noMD and that of MD mice did not differ between each other (one-way ANOVA, post-hoc Holm-Sidak method, p = 0.417).

### Proteomic identification of proteins modulated by fluoxetine in the adult mouse visual cortex

**Figure 2:**
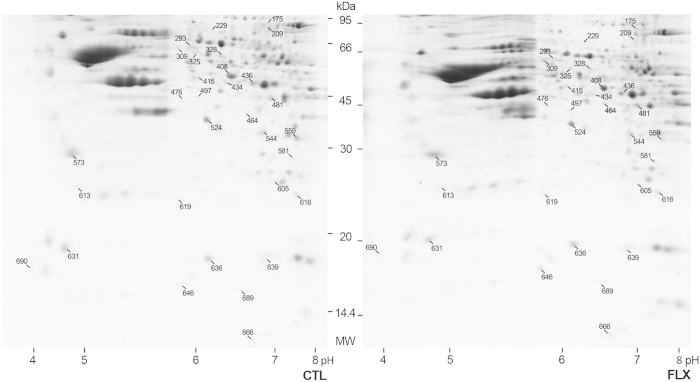
Fluoxetine modulates the proteomic profile in the adult mouse visual cortex. Representative gels showing the 2D profile of the visual cortex of fluoxetine-treated (FLX) and age-matched (CTL) adult mice, stained with colloidal Coomassie Brilliant Blue G250. First dimension was performed loading 60 μg of whole protein extracts from visual cortical samples on immobilized pH 3–10 nonlinear gradient strips. Second dimension was performed on 12% SDS-PAGE gels. The 31 spots accepted as significantly differentially expressed between the two experimental conditions are indicated by a line and the corresponding match IDs (see Table 1 and Table 2). These spots were manually excised and analyzed by mass spectrometry. MW, molecular weight (kDa). To improve the clarity of the presentation gel images were cropped. Full-length uncropped images of the same gels are presented in Supplementary Figure F1.

In order to identify possible molecular candidates for the fluoxetine-induced effect of restoration of enhanced levels of plasticity in the adult mouse visual cortex, we used 2D differential gel electrophoresis followed by mass spectrometry on visual cortical samples from fluoxetine-treated (n = 6) and age-matched controls (n = 6). Image analysis of the gels showed a similar number and pattern distribution of spots in the twelve gels (see representative gel in Fig. 2), with minor differences possibly due to artifacts of the methodology. Between 511 and 614 spots were counted on each gel with an average number of 563 spots per gel. A total of 450 spots were successfully matched across the 12 gels corresponding to approximately 80% of the counted spots. In order to evaluate protein level differences between gels, the relative volume parameter (%Vol) was used, which is a rather efficient measure as it takes into account variations due to protein loading and staining, by considering the total volume over all the spots in the gel. Thirty-one of the analyzed spots were accepted as significantly differentially expressed between the two experimental conditions (see Materials and Methods for details). These spots were manually excised, in gel digested with trypsin and analyzed with a 4800 MALDI TOF/TOF Analyzer Mass spectrometer (Abi Sciex).

Mass spectrometry gave sufficient information to identify proteins present within 24 of the 31 spots (77% success rate of the methodology). In these 24 spots, a single protein was identified per spot, resulting exactly in a total of 24 successfully identified and differentially expressed proteins. In the remaining spots no protein was identified, possibly because of keratin contamination (one spot) or because not enough protein material was present (six spots). A complete list of the spots with statistically significant changes in level is reported in Table 1 and Table 2. Details of the mass spectrometry analysis are reported in Supplementary Table S1 and S2.

The analysis indicated that sixteen of the identified proteins were upregulated in the visual cortex of fluoxetine-treated adult mice with an increase ranging from approximately 17% to more than 90% (as calculated by the average difference ratio in the %Vol value), while eight were downregulated with a decrease ranging from approximately 13% to more than 30%.

**Figure 3:**
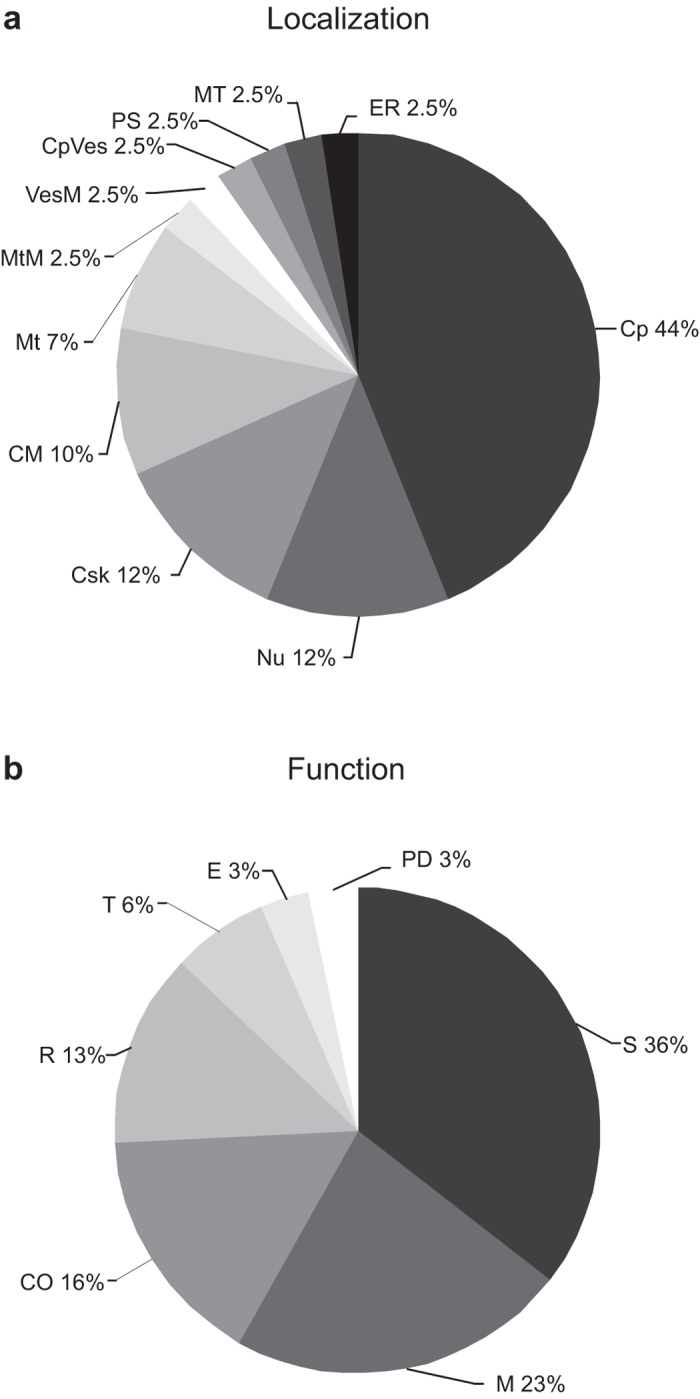
The identified proteins were assigned to eleven different cellular/subcellular localizations and to seven different functional processes by using the Protein Knowledgebase UniProtKB. Pie charts represent the percentage of each localization (**A**) or functional (**B**) process entry with respect to the total number of entries. Same abbreviations as in Table 1.

Molecular weight and isoelectric point theoretical values of the 24 proteins were obtained in the Protein Knowledgebase UniProtKB and compared to the corresponding values calculated on the gels using the Melanie 6.0 software. The comparative analysis revealed no mayor discrepancy between the theoretical and experimental values of both the *pI* and MW of the identified proteins. By using the Protein Knowledgebase UniProtKB the 24 different identified proteins were assigned to a total of 11 different cellular/subcellular localizations (see Material and Methods for details), with the same protein possibly being assigned to more than one single localization (total of 41 entries). This analysis revealed that most of the entries corresponded to Cytoplasm localization (18 entries, 44% of total entries), 5 to the Nucleus (12%), 5 to the Cytoskeleton (12%), 4 to Cell Membrane (10%), 3 to Mitochondrion (7%), and 1 (2.5%) to Mitochondrial Membrane, Vesicular Membrane, Cytoplasmic Vesicle, Proteasome, Microtubule and Endoplasmic Reticulum (see Fig. 3A). Considering their biological function, the proteins were classified as belonging to a total of 7 main functional processes (total of 31 entries). The analysis revealed that most of the entries corresponded to Signaling processes (11 entries, 36% of total entries), 7 to Metabolism (23%), 5 to Cytoskeleton Organization (16%), 4 to Redox (13%), 2 to Transport (6%), and 1 (3%) to Endocytosis and Protein degradation (see Fig. 3B).

### Validation of 2D results by western blot analysis

**Figure 4:**
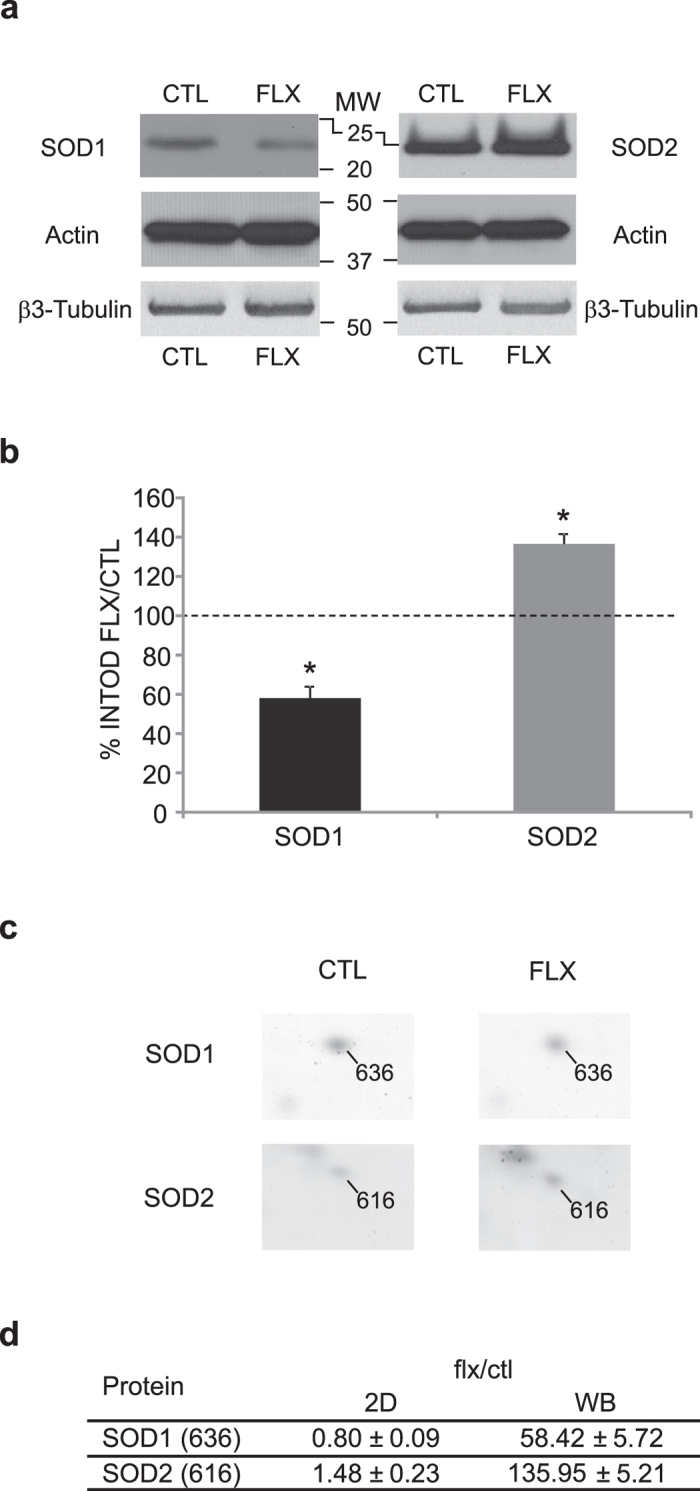
Validation of 2D gels protein level differences by western blot analysis. **A**) Representative western blot of visual cortical samples from fluoxetine-treated (FLX) and age-matched controls (CTL) incubated with anti-SOD1, anti-SOD2, anti-actin or anti-β3-tubulin antibodies. Antibodies recognized a main band at 20–22 kDa (SOD1), 25kDa (SOD2), 42 kDa (actin) or 55 kDa (β3-tubulin). To improve the clarity of the presentation blot images were cropped. Larger images of the same blots are presented in Supplementary Figure F2. **B**) The SOD1 and SOD2 INTOD values were normalized to the corresponding actin value, and fluoxetine-treated values normalized to the corresponding value in control samples. SOD1 protein level decreased of approx. 40% in the visual cortex of fluoxetine-treated mice when compared to controls (SOD1: INTOD flx/ctl = 58.42 ± 5.72, Mann-Whitney U test, p < 0.05, n = 6), while SOD2 protein level increased approx. 35% (SOD2: INTOD flx/ctl = 135.95 ± 5.21, n = 6, Mann-Whitney U test, p < 0.05). Normalization against β3-tubulin values did not differ from what obtained with actin (SOD1: INTOD flx/ctl = 64.66 ± 13.54, n = 4, Mann-Whitney U test, p < 0.05; SOD2: INTOD flx/ctl = 141.13 ± 6.96, n = 4, Mann-Whitney U test, p < 0.05). **C**) Detail of the 2D gel electrophoresis indicating the spots corresponding to SOD1 (636) and SOD2 (616) in fluoxetine-treated (FLX) and age-matched controls (CTL). **D**) The table reports the amount of modulation of SOD1 and SOD2 protein level by fluoxetine as calculated in the 2D gel image analysis (2D gel) and in the western blot analysis (WB), with corresponding S.E.M.

In order to validate protein level differences observed in 2D gels, we used western blot analysis for a quantification of two of the previously identified proteins, SOD1 and SOD2, which were chosen as representative of a down and an upregulation by fluoxetine, respectively. As shown in Fig. 4, western blot analysis was performed with specific antibodies which detected a main band at 20–22 kDa for SOD1 and at 25 kDa for SOD2. The intensity of the bands was normalized to the house-keeping gene actin and then the value obtained in fluoxetine-treated samples was normalized to the corresponding value in control samples. Semi-quantitative analysis of the western blot confirmed the tendency observed in the 2D gel image analysis. In details, SOD1 protein level decreased of approx. 40% in the visual cortex of fluoxetine-treated mice when compared to controls (SOD1: INTOD flx/ctl = 58.42 ± 5.72, Mann-Whitney U test, p < 0.05, n = 6), while SOD2 protein level increased approx. 35% (SOD2: INTOD flx/ctl = 135.95 ± 5.21, n = 6, Mann-Whitney U test, p < 0.05). As a further control, experiments were repeated using β3-tubulin as normalization value, and the results obtained were in agreement with what shown using actin (SOD1: 64.66 ± 13.54, n = 4, Mann-Whitney U test, p < 0.05; SOD2: INTOD flx/ctl = 141.13 ± 6.96, n = 4, Mann-Whitney U test, p < 0.05).

## Discussion

In the present work we analyzed the effects of a long-term treatment with fluoxetine on visual cortical plasticity in adult mice and studied potential candidates mediating fluoxetine effects by means of 2D gel electrophoresis followed by mass spectrometry.

Obtained data clearly revealed that long-term fluoxetine treatment restores OD plasticity in the adult mouse visual cortex. This effect is similar to what previously observed in the visual cortex of adult rats treated with fluoxetine^4^. With the aim of identifying possible mediators of the fluoxetine-induced reopening of adult cortical plasticity, we looked for differentially expressed proteins using a 2D gel electrophoresis differential approach. On average, 563 spots were counted on each gel, which is obviously only a fraction of the 10000 or more proteins actually expressed in the mammalian brain tissue. Nevertheless, we were able to identify at least 24 proteins which were modulated in the adult mouse visual cortex by long-term fluoxetine treatment. The relatively low number of differentially expressed proteins in the experimental conditions analyzed is not surprising. In fact, similar studies using larger strips with narrower pH gradients, or more sophisticated methodologies such as the DIGE, prefractionation by RP-HPLC, and the isobaric tag for relative and absolute quantitation (iTRAQ), have identified a number of differentially expressed proteins in a similar range^8^,^9^,^10^,^11^.

We identified sixteen proteins that were upregulated and eight that were downregulated in the visual cortex of fluoxetine-treated adult mice. We confirmed by western blot analysis the modulation of the level of two differentially expressed proteins, SOD1 and SOD2, representative of the down and upregulation by fluoxetine in the 2D gel analysis.

The amount of variation in the level of the protein identified between the two experimental conditions ranged from 15% to 90%. While we expected a higher percentage range of modulation induced by fluoxetine, this result is not surprising. In fact previous experiments in the cat and rat visual cortex during development showed that only a few proteins present a manifold difference^8^,^9^,^10^,^12^. By using a more quantitative methodology in the mouse visual cortex the average difference observed during development was between 10 and 40% when analyzed following other experimental approaches, as the modulation of the visual input by MD or dark rearing^11^.

An original result of the present proteomic approach is the identification of proteins which are modulated in the visual cortex of adult mice by the long-term treatment with fluoxetine. A few of the proteins identified in the present study have been identified also in other previously published works studying by proteomics the effects of fluoxetine and other antidepressants on different brain areas *in vivo* and *in vitro*^13^,^14^. However, for consistency, the present discussion is limited to data obtained mainly in the visual cortex. By using another large-scale approach, a previous work analyzed the regulation of gene expression by fluoxetine in the rat visual cortex by DNA microarray^6^. Among the 197 genes identified in this previous study, only one coincides with proteins identified in our study, the PDIA3 protein. This may be due to differences in the methodologies used and the species used. Moreover, it is known that RNA abundances only partially predict protein abundances as the two molecular species differ in stability, synthesis levels, mechanisms of degradation and regulation^15^. Finally, also another work reported the absence of a strict correlation between transcriptomic and proteomic experimental approaches performed in the cat visual cortex^8^.

### Relevance of the identified proteins in controlling plasticity processes

The main aim of the present work was to contribute to the identification of proteins which may mediate the reopening of cortical plasticity induced by fluoxetine in the adult mouse visual cortex. Through a bioinformatic analysis, the identified proteins were assigned to various biological processes and these are now discussed in light of their potential relevance for the regulation of plasticity.

#### Cytoskeleton organization

It is well known that cortical plasticity is associated with a rewiring of circuits at axon and dendritic spine level^16^. Here we showed that fluoxetine upregulates the level of five proteins which are directly involved in the control of cytoskeleton organization: ARP2, TCTP, CDC42, PROF2 and DYL2. ARP2 (through the ARP2/3 complex), CDC42 and PROF2 interact with actin filaments, modulating their branching and the composition of the final network. Increasing evidence showed a direct interaction among these three proteins in the control of morphological plasticity of dendritic spines^17^. CDC42 belongs to the family of small Rho GTPases known to modulate various intracellular signaling pathways. It is worth noting that the pharmacological persistent activation of Rho GTPases in the visual cortex of adult rats triggers structural remodeling and functional plasticity^18^. As for DYL2 and TCTP, these proteins interact with microtubules, modulating their stability and playing a role on distinct synaptic processes^19^. Moreover, it is known that fluoxetine plays a relevant role in the long term structural remodeling of synaptic contacts in different areas^20^ including the adult mouse visual cortex^21^.

Together with the observation that these proteins have been associated to the regulation of plastic processes in the hippocampus and forebrain - from the maturation and development of dendritic spines and synapses to the control of LTP and LTD^22^ - it is likely that they are part of the molecular machinery mediating the plastic structural modifications induced by fluoxetine. In agreement with this concept, a recent proteomic work on the mouse visual cortex showed a modulation of CDC42, PROF2, DYL2 and another subunit of the ARP2/3 complex in which ARP2 participates (ARPC4B) by sensory experience and during development, suggesting a potential role in cortical plasticity^11^.

#### Endocytosis and transport

We found an upregulation by fluoxetine of the proteins NECP1 and DYL2. NECP1 directly participates in the clathrin-dependent endocytosis in association to other proteins (as AP1G1 and AP2A1) components of the adaptator complexes AP-1 and AP-2^23^. It has been shown that NECP1 regulates the neuronal endocytosis of AMPARs and GABARs at postsynaptic level, and the vesicle recycling in the presynapsis^23^, thus playing a role on plastic processes through modulation of synaptic transmission^24^,^25^. A previous work on the mouse visual cortex showed a modulation during development and by visual experience of AP2A1, a protein interacting with NECP1 in the formation of the AP-2 complex^11^.

Considering DYL2, besides its role on cytoskeleton organization (see above), it is a component of the dynein complex acting on intracellular retrograde motility of vesicles and organelles along microtubules also at the synaptic level^19^. It has been shown that DYL2 is directly involved in the transport of BDNF, some matrix metalloproteinases, neuroligin, PSD-95 and GABARs^26^,^27^,^28^, and thus in the control of LTP and LTD in the hippocampus^29^. Moreover, DYL2 is modulated by visual experience in the visual cortex of mice^11^.

It has to be underscored that molecules shown to be internalized by NECP1 and those transported by DYL2 are fundamental for determining the excitation/inhibition balance in the cortex (AMPARs, GABARs, BDNF), and for contributing to define the extracellular biochemical milieu (metalloproteinases), two processes fundamental for plasticity regulation in the visual cortex^30^.

#### Protein degradation

We found that fluoxetine treatment upregulates the level of the PSA2 protein in the adult mouse visual cortex. PSA2 is a component of the proteasome, a key proteolytic enzymatic system governing the degradation of the majority of intracellular proteins. It is interesting to note that the proteasome function has been associated to activity-dependent neuronal signaling processes and to the regulation of synaptic transmission^31^. A recent study reported that voluntary physical exercise may engage proteasome function to benefit the brain after trauma associated with long-term decrements in synaptic plasticity and cognitive function^32^. This is relevant considering the effects induced by the experimental protocol of enriched environment (EE) on restoring plasticity in the visual cortex of adult rodents^2^, where physical exercise is a major component. The molecular mechanisms inducing the reopening of plasticity identified to date are very similar between EE and fluoxetine treatment^2^,^4^,^7^. Thus, we may suggest that, similarly to EE, fluoxetine could increase adult cortical plasticity acting also at protein degradation level through PSA2 or other analogous components.

#### Control of redox state

We found that fluoxetine modulates the level of four proteins involved in the control of cellular redox state (three were up- and one downregulated). SOD1 and SOD2 are involved in the oxidative metabolism, eliminating free radicals from the cytoplasm and mitochondria, and defects in the SOD1 gene are associated with familial forms of amyotrophic lateral sclerosis^33^. SOD1 and SOD2 have been shown to increase in the cat and rat visual cortex during development^10^,^12^. PDIA3 possesses an oxido-reductase domain which participates in the folding of proteins (chaperon activity), and its level is modulated during the critical period in the rat and cat visual cortex^10^,^12^ and by visual experience in the mouse^11^. Besides several other metabolic processes, the SERA protein acts also as an oxido-reductase in the biosynthesis of aminoacids, neurotransmitters and nitric oxide^34^.

It is interesting to note that the control and regulation of the intracellular redox homeostasis has been directly involved in several plasticity processes in various brain areas^35^. Finally, it has been shown in different experimental models that fluoxetine and other anti-depressants may modulate anti-oxidant cellular defenses^36^. Thus it is possible that the four proteins modulated by fluoxetine participate of a redox control mechanism which may regulate cortical plasticity levels.

#### Intracellular signaling

Here, we showed that fluoxetine treatment modulates in the adult mouse visual cortex the level of eleven proteins (nine up- and 2 downregulated) which play fundamental roles in the control of various intracellular signaling processes.

A fundamental and well characterized role in the control of cortical plasticity is played by the kinases ERK1/2, PKA and CaMKII^37^, and the phosphatase calcineurin^38^. In the present work we showed that fluoxetine increases the level of CALM1 - a calcium binding small regulatory protein that participates in the control of a large number of enzymes, including CaMKII, and the level of PTPA - an activator of the serine/threonine protein phosphatase 2A whose activity has been related to LTP and LTD in the hippocampus and cerebellum^39^,^40^, and the activation of silent synapses^41^, while its potential role in the visual cortex has not been studied yet. In agreement with our observation, a previous work showed that CALM1 increases during the critical period of plasticity in the mouse visual cortex and is modulated by visual experience^11^. Another kinase modulated by fluoxetine was NDKA, which possesses different kinds of activity, and is necessary for neuronal development and cell fate^42^, but whose potential role on plastic processes is unexplored.

Two of the identified proteins have been shown to interact with and modulate neuronal receptors. One is HINT1, a protein enriched in the CNS which regulates the interaction between NMDA and opioid receptors^43^. Opioid receptors are expressed in the visual cortex and have been shown to modulate cortical GABAergic responses, thus influencing the inhibitory tone and eventually plastic processes^44^. The other is 1433Z, which is part of a 14-3-3 group of proteins abundantly expressed in the mammalian brain, binding different proteins acting on cellular cycle, transcription control, signaling transduction, intracellular trafficking and ionic channel regulation^45^. Particularly relevant is the observation that 1433Z level is modulated in different experimental models of schizophrenia and of cocaine abuse^46^, and the demonstration that 1433Z is acutely required for learning and memory, embryonic development and behavioral neuroplasticity in *C. elegans*^47^. 1433Z is modulated by visual experience in the visual cortex of mice^11^, while in rats and cats other isoforms are highly expressed during the critical period^10^,^12^.

GLNA is a diffuse enzyme in the CNS regulating the glutamine-glutamate cycle, thus playing a fundamental role in the control of excitability of neurons and astroglia. Modulation of this enzyme and the consequent alterations of the glutamate homeostasis have been associated to several neurological disorders as epilepsy, autism, Down syndrome and Alzheimer disease^48^,^49^,^50^. In the cat visual cortex, this enzyme has been shown to increase its level during development^8^,^10^.

Another modulated protein turned out to be VDAC1, which has been shown to be regulated during development and by visual experience in the cat and the mouse visual cortex, together with other forms, VDAC2 and VDAC3^10^,^11^. VDAC1 belongs to the family of pore-forming small proteins inserted in the mitochondria external membrane and in the plasma membrane of all eukaryotes. These proteins are considered important in the buffering of calcium in mitochondria localized in the synapses, and thus acting as regulators of signaling and synaptic efficacy^51^.

Finally, we found an upregulation of SYUA, a synaptic protein richly expressed in the CNS and involved in various neurodegenerative diseases, collectively known as synucleinopathies (Alzheimer’s and Parkinson’s disease, multiple system atrophy, dementia with Lewy bodies). SYUA is abundantly expressed in presynaptic terminals and associated with synaptic vesicles, and several studies have revealed an involvement of this protein in synaptic vesicle recycling, neurotransmitter synthesis and release, and synaptic plasticity^52^.

Other proteins modulated by fluoxetine were CDC42 and TCTP (see cytoskeleton organization), and ESTD (see metabolism).

#### Metabolism

Several of the identified proteins modulated by fluoxetine were classified as playing a role in various metabolic processes, including the amino acid and NTPs synthesis (GLNA, SERA, and NDKA), the detoxification via hydrolase activity (ESTD) and the glycolisis for energy production (ENOA, ALDOC, MDHC). In agreement with our results, it has been reported that MDHC is modulated in the visual cortex during development^10^,^11^,^12^, and, in the hippocampus, by a GABAR-induced plasticity process^53^. In the present context it is interesting to note that the expression and activity of the MDHC protein is modulated by caloric restriction, which in turn is known to benefit the ageing brain^54^. Caloric restriction has received particular attention in the last years as a potential non invasive therapy for the functional recovery of different disorders on the CNS. Interestingly, it has been recently shown that this experimental protocol restores high levels of plasticity in the visual cortex of adult rats^55^, suggesting that caloric restriction and fluoxetine administration may share final metabolic processes, such as regulation of MDHC levels.

Here, we reported that a long-term treatment with the anti-depressant fluoxetine is able to restore high levels of plasticity in the adult visual cortex also in mice. Together to previous results^21^, this observation may open the way to further investigations on the cellular and molecular mechanisms involved in the fluoxetine induced potentiation of plasticity in this species, by the employment of genetically modified experimental animals.

We exploited the potential of 2D gel electrophoresis followed by mass spectrometry for the identification of protein level differences between different experimental conditions. This or similar proteomic approaches, to date underexploited in the field of experience-dependent plasticity, may provide new insights into the molecular mechanisms regulating brain plasticity.

In the last years, many efforts have been done to understand the general mechanism underlying fluoxetine action. While comparison of data is hindered by the differences in the strategies used in experimental models (as type of antidepressant, dose, times, method of application, etc.), a few general mechanism have been identified. It has been proposed recently that modulation of neuronal plasticity, including neurogenesis, growth and retraction of axonal and dendritic branches, control of synaptic connections and plasticity of synaptic strength may be a target of antidepressants action^56^. Thus, the use of the visual cortex, a well-characterized model for cortical development and plasticity, is particularly relevant, as it is widely thought that similar processes govern the development and tuning of neuronal connectivity in other cortical areas. It is interesting to note that nine of the proteins identified in our work (involved in the control of cytoskeleton organization, redox state, signaling and metabolism), have been shown to be modulated by fluoxetine or other antidepressants also in the frontal cortex and hippocampus^13^,^14^, suggesting possible common mechanisms of antidepressants action in the central nervous system. However, several important questions remain to be addressed before these general principles can be extended to higher cortical regions.

In conclusion, we showed an association between fluoxetine-induced reactivation of visual cortex plasticity and the modulation of proteins involved in various biological processes possibly affecting brain plasticity. Further study will target these proteins as promising candidates for the control of visual cortical plasticity in the adult brain.

## Materials and Methods

### Animals and treatment

All experiments were performed in accordance with the approved guidelines and regulations of the Uruguayan and Italian Animal Research Ethic Committees. The “Comisión Nacional de Experimentación Animal”, Uruguay, and the Ministry of Public Health, Italy, approved all the experimental protocols used. A total of 41 C57BL6/J adult mice were used in the experiments (both sexes, housed 6/cage). Fluoxetine treatment followed drug concentrations and schedules previously used^57^. In details, adult mice were exposed for 4 weeks, from postnatal day (P) 70, to oral fluoxetine (Fluoxetine-hydrochloride, Galeno, Italy or Selectchemie, Laboratorios Gador S.A., Uruguay) dissolved in tap water (0.1 mg/ml), or to a normal water drinking regimen (controls). Solutions were prepared fresh twice a week (see details in Supplementary Table S3). A 3 days monocular deprivation (MD) was performed as previously reported^58^, during the last three days of fluoxetine treatment. For proteomic and western blot experiments, mice were sacrificed by cervical dislocation, right and left visual cortices dissected under a stereoscopic microscope on ice in saline solution (NaCl 0.9%), pooled together and stored at −80 °C for further processing.

### Electrophysiology

Recordings were performed as previously described^59^, on naive (noMD), monocularly deprived untreated (MD) and monocularly deprived fluoxetine-treated (flxMD) adult mice. Mice (n = 17), were anesthetized i.p. with Zoletil-100 (40 mg/kg, Virbac), and Xilor (10 mg/kg, Sigma), and placed in a stereotaxic frame. After exposure of the brain surface, a micropipette filled with NaCl 3 M was inserted into the cortex 2.8–3.2 mm from λ point. The eyes were fixed and kept open by means of adjustable metal rings surrounding the external portion of the eye bulb. To record visual evoked potentials (VEPs), the electrode was advanced at a depth of 100 or 400 μm within the cortex, where VEPs had their maximal amplitude. Signals were band-pass-filtered (0.1–100 Hz), amplified, and fed to a computer for analysis. At least 50 events were averaged in synchrony with the stimulus contrast reversal. Transient VEPs in response to abrupt contrast reversal (1 Hz) were evaluated in the time domain by measuring the peak-to-baseline amplitude and peak latency of the major positive (at 100 μm depth) or negative (at 400 μm depth) component. Visual stimuli were horizontal sinusoidal gratings of different spatial frequencies, generated by a VSG2/2 card running custom software and presented on a monitor (20 × 22 cm; luminance 15 cd/m^2^) positioned 20 cm from the mouse eyes. Binocularity was assessed calculating the contralateral to ipsilateral (C/I) VEP ratio at 0.05 cycles per degree (c/deg), i.e., the ratio of VEP amplitudes recorded by stimulating the contralateral and ipsilateral eye with respect to the brain side where recording is performed. For each animal, at least 12 independent C/I VEP ratio values were calculated and averaged together from three well-spaced traces along the antero-posterior axis of V1. Care was taken to equally sample VEPs across the two cortical depths so that all layers contributed to the analysis.

### 2D-PAGE

2D-PAGE experiments were performed on adult untreated mice (CTL) and adult fluoxetine-treated mice (both without monocular deprivation). Proteins were extracted from visual cortical samples by mechanical homogenization and sonication in lysis buffer (7 M urea, 2 M thiourea, 4% w/v CHAPS, 56 mM DTT, protease inhibitor cocktail, Sigma). The supernatant was cleaned with 2D Clean-Up kit (Amersham) and resuspended in lysis buffer (without PI). Protein concentration was measured by the mini-Bradford assay at λ = 595 nm. Isoelectrofocusing (IEF) was performed on an Ettan IPGphor II System, applying 60 μg of sample on immobilized pH 3–10 nonlinear gradient strips (Amersham). Prior to IEF, strips where passively rehydrated for 18 hours at 20 °C. Focusing was initiated at 300 V and the voltage gradually increased to 5000 V within 2 h and 20 min and kept constant for 1 h, for a total of 8574 Vh. After IEF, strips where equilibrated twice for 15 min in a solution containing 50 mM Tris pH 8.8, 6 M urea, 30% (v/v) glycerol and 2% (w/v) SDS. DTT (1%, w/v) was added to the first, and iodoacetamide (2.5%, w/v) to the second equilibration step. IPG strips were then placed on top of a 1 mm thick SDS polyacrylamide gel (12% T; 2.67% C) and run in sets of 2 at 10 mA/gel for 10 min and then at 20 mA/gel. Proteins were then fixed with 10% acetic acid, 40% ethanol in water for 30 min and gels stained with colloidal Coomassie Brilliant Blue G250 (Affymetrix, US) for 48 hs. Molecular masses were determined by running standard protein markers (Precision Plus Protein™ Unstained Standards, BioRad), and *pI* values were used as given by the supplier of the IPG strips. Gels were destained with H_2_O and scanned in UMAX PoweLook 1120 scanner using the LabScan 5.0 software (Genebio Amersham Biosciences).

### Gel image analysis

Gel images were analyzed using the Melanie 6.0 software (Genebio Amersham Biosciences). Spots were automatically detected and edited manually to improve accuracy. Landmarks (8–10/gel) were established from all regions of the gels and assigned only when a spot was clearly present in all gels. Matching was automatically obtained and manually checked. The relative volume parameter (%Vol) was used for evaluating protein level differences between gels. The %Vol value of spots in fluoxetine-treated samples was normalized to the corresponding value in the control sample and obtained data were analyzed for statistical differences using the Past 2.14 free analysis system^60^ with Mann-Whitney U test with p < 0.05 as threshold for significance.

### Mass Spectrometry

Protein spots selected for identification by peptide mass fingerprint plus MS/MS analysis of selected peptides were manually excised from the gel and stored at 4 °C. Spots were destained in 100 μl μof a solution containing 50% (vol/vol) 0.2 M ammonium bicarbonate pH 8.0 and acetonitrile three times for 30 min at 30 °C in a thermomixer at 1400 rpm and finally air dried. Proteolytic in-gel digestion was carried out with 10 μl of sequencing-grade trypsin (Promega, 0.1 μg/μl in 67 mM ammonium bicarbonate pH 8.0) at 37 °C overnight. Peptides were then extracted from gels with 100 μl of a solution containing 60% acetonitrile in 0.1% trifluoroacetic acid, concentrated by vacuum drying, and desalted with commercial reverse-phase microcolumns C18 (OMIX Pipette tips, Varian). Peptides were eluted directly on the spectrometer plate with 2 μl of matrix solution (α-cyano-4-hydroxycinnamic acid, CHCA in 60% acetonitrile, 0.1% trifluoroacetic acid). Mass spectrometry measurements were carried out in a MALDI-TOF/TOF system (4800 MALDI TOF/TOF Analyzer AB Sciex), and spectra acquired in reflector mode and internally calibrated with autolytic fragments of trypsin. MS/MS analysis of selected *m/z* values was performed in order to increase the confidence of the identification. Proteins were identified by database searching (NCBIir 2013/05/30) with *m/z* values obtained in MS and MS/MS acquisition modes using the MASCOT program (Matrix Science, www.matrixscience.com) in the Sequence Query search mode. Search parameters were: up to one trypsin miscleavages allowed; cysteine carbamidomethylation and methionine oxidation as variable modifications; mass tolerance of 0.08 and 0.35 Da for precursor and fragment ions respectively. Proteins with significant mascot scores and at least one peptide sequences confirmed by MS/MS were considered positively identified (p < 0.05).

### Bioinformatics

Once proteins were identified by database searching with the MASCOT program, more information was searched in the Protein Knowledgebase UniProtKB (www.uniprot.org) with the corresponding accession number. For localization and biological process, the Gene Ontology search was used, including evidences obtained by Traceable Author Statement, Inferred from Direct Assay, Expression Pattern, Mutant Phenotype, Physical Interaction, and Genetic Interaction. Relation with specific processes was investigated using the National Center for Biotechnology Information (NCBI) database (www.ncbi.nlm.nih.gov).

### Western Blot

Western blot experiments were performed as previously described^61^. The same amount of protein per sample (30 μg) was electrophoresed in a 15% sodium dodecyl sulfate polyacrylamide gel (SDS-PAGE) at 160 V for one hour. Transfer onto nitrocellulose membrane (GE Healthcare) was performed at 100 V for two hours. In each experiment two gels were run and loaded with the same samples: one gel for analysis with a rabbit polyclonal anti-SOD1 antibody^62^ and the second with a mouse monoclonal anti-SOD2 antibody (Santa Cruz Biotechnology, CA, USA). Following blocking (5% non-fat dry milk in Phosphate Buffer Saline solution, PBS) for 1 h at RT and blots were probed overnight at 4 °C with anti-SOD1 (1:2000) or anti-SOD2 (1:2000) primary antibody in PBS containing 0.05% Tween 20 (PBST). Blots were then incubated with horseradish peroxidase (HRP)-linked anti-rabbit (Sigma, 1:5000) or anti-mouse (Sigma, 1:10000) secondary antibody for 1 h at RT. Immunoreactive bands were visualized using enhanced chemioluminescence system (Amersham) with x-ray films (Agfa) or using the GBOX ChemiSystem tool (SynGene). Membranes were then stripped (5 min, NaOH 0.1 M), washed in PBST, blocked in 5% non-fat dry milk in PBS and reincubated with the polyclonal rabbit anti-actin antibody (1:2000, Sigma) or the monoclonal mouse anti-β3-tubulin antibody (1:1000, Cell Signaling).

The bands were quantified using the NIH Image J 1.46r free analysis system, using the integrated optical density (INTOD) as index of the signal. The INTOD of SOD1 or SOD2 was normalized to the corresponding actin value in the same sample and the data of fluoxetine-treated samples normalized to the age-matched control values. As a further control, experiments were repeated using β3-tubulin as normalization value. A total of 12 mice (n = 6 fluoxetine-treated and n = 6 controls) were used in western blot experiments. To control inter-experimental variability each experiment was repeated 2–3 times. All data were analyzed by using with Mann-Whitney U test with p < 0.05 as threshold for significant difference. Statistical analysis of data was carried out using Past 2.14 free analysis system^60^.

### Animal Research Ethic Statement

 All experiments were performed in accordance with the approved guidelines and regulations of the Uruguayan and Italian Animal Research Ethic Committees (“Comisión Nacional de Experimentación Animal”, Uruguay, and the Ministry of Public Health, Italy) which approved all the experimental protocols used. 

## Additional Information

**How to cite this article**: Ruiz-Perera, L. *et al.* Fluoxetine increases plasticity and modulates the proteomic profile in the adult mouse visual cortex. *Sci. Rep.* **5**, 12517; doi: 10.1038/srep12517 (2015).

## Supplementary Material

Supplementary Information

## Figures and Tables

**Table 1:** Proteins with higher level in fluoxetine-treated samples.

**Spot N°**	**Protein accession**	**Full name**	***pI***	**MW (Da)**	**Flx/Ctl** **±** **SEM**	**Localization**	**Function**
464	ARP2_MOUSE	Actin-related protein 2	6,31	44601	1,91 ± 0,53	Csk	CO
646	PROF2_MOUSE	Profilin-2	6,55	15022	1,64 ± 0,23	Cp/Csk	CO
209	No ID	No ID	—	—	1,63 ± 0,20	—	—
497	PTPA_MOUSE	Serine/threonine-protein phosphatase 2A activator	5,95	36575	1,58 ± 0,19	Cp/Nu	S
690	CALM_MOUSE	Calmodulin	3,9	16838	1,58 ± 0,17	CP/Csk	S
175	No ID	No ID	—	—	1,56 ± 0,16	—	—
613	TCTP_MOUSE	Translationally-controlled tumor protein	4,76	19450	1,54 ± 0,24	Cp	S/CO
476	NECP1_MOUSE	Adaptin ear-binding coat-associated protein 1	5,97	29621	1,48 ± 0,19	VesM/CM	E/T
616	SODM_MOUSE (SOD2)	Superoxide dismutase [Mn], mitocondrial	8,8	24662	1,48 ± 0,23	Mt	R
605	PSA2_MOUSE	Proteasome subunit alpha type-2	6,92	25910	1,43 ± 0,09	Cp/Nu/PS	PD
415	No ID	No ID	—	—	1,42 ± 0,15	—	—
666	DYL2_MOUSE	Dynein light chain2, cytoplasmic	6,81	10343	1,42 ± 0,10	Cp/Csk/MT	CO/T
309	PDIA3_MOUSE	Protein disulfide-isomerase A3	5,88	56643	1,39 ± 0,19	ER	R
581	DHPR_MOUSE	Dihydropteridine reductase	7,67	25554	1,38 ± 0,22	Cp	R
619	CDC42_MOUSE	Cell division control protein 42 homolog isoform 2	5,76	21297	1,37 ± 0,11	CM/Cp/Csk	S/CO
559	VDAC1_MOUSE	Voltage-dependent anion-selective channel protein 1	8,62	30737	1,35 ± 0,17	CM/MtM	S
544	ESTD_MOUSE	S-formyl glutathione hydrolase	6,7	31299	1,27 ± 0,08	Cp/CpVes	S/M
573	1433Z_MOUSE	14-3-3 protein zeta/delta	4,72	27708	1,26 ± 0,12	Cp	S
436	GLNA_MOUSE	Glutamine synthetase	6,64	42161	1,17 ± 0,05	Cp/Mt	M/S

Mass spectrometry identification of differential V1 protein levels induced by fluoxetine. The table reports the list of the 19 spots with higher level in fluoxetine-treated samples (as indicated in Fig. 2) with corresponding spot number, SwissProt protein accession number, full name, theoretical *pI* and MW (Da), the average normalized spot %Vol ratio between fluoxetine-treated and age-matched control samples with corresponding S.E.M., the main localization and indication of main biological processes in which the given protein is known to take part. Details of the mass spectrometry analysis are reported in Supplementary Table S1. Protein Knowledgebase UniProtKB was used to obtain MW and *pI* theoretical values, the main localization (Cp, Cytoplasm; Nu, Nucleus; Csk, Cytoskeleton; CM, Cell Membrane; Mt, Mitochondrion; MtM, Mitochondrial Membrane; VesM, Vesicular Membrane; CpVes, Cytoplasmic Vesicle; PS, Proteasome; MT, Microtubule; ER, Endoplasmic Reticulum), and the main biological function (S, Signaling; M, Metabolism; CO, Cytoskeleton Organization; R, control of redox state; T, Transport; E, Endocytosis; PD, Protein Degradation).

**Table 2:** Proteins with lower level in fluoxetine-treated samples.

**Spot N°**	**Protein accession**	**Full name**	***pI***	**MW (Da)**	**Flx/Ctl** **±** **SEM**	**Localization**	**Function**
229	No ID	No ID	6,36	13768	0,67 ± 0,08	Cp/Nu	S
689	HINT1_MOUSE	Histidine triad nucleotide-binding protein 1			0,68 ± 0,09		
293	No ID	No ID			0,70 ± 0,05		
325	No ID	No ID			0,70 ± 0,06		
481	ALDOC_MOUSE	Fructose-bisphosphate aldolase C	6,47	39307	0,74 ± 0,05	Mt	M
328	SERA_MOUSE	D-3-phospho glycerate dehydrogenase	6,12	56549	0,75 ± 0,05	Cp	M
434	No ID	No ID			0,80 ± 0,05		
631	SYUA_MOUSE	Alpha-synuclein	4,74	14476	0,80 ± 0,03	Cp	S
636	SODC_MOUSE (SOD1)	Superoxide dismutase [Cu-Zn]	6,23	15955	0,80 ± 0,09	Cp/Nu	R
639	NDKA_MOUSE	Nucleoside diphosphate kinase A	8,44	18672	0,83 ± 0,06	Cp/Nu	M/S
408	ENOA_MOUSE	Alpha-enolase	6,37	47111	0,86 ± 0,04	Cp/CM	M
524	MDHC_MOUSE	Malate dehydrogenase, cytoplasmic	6,16	36454	0,87 ± 0,05	Cp	M

Mass spectrometry identification of differential V1 protein levels induced by fluoxetine. The table reports the list of the 12 spots with lower level in fluoxetine-treated samples (as indicated in Fig. 2). Same abbreviations as in Table 1. Details of the mass spectrometry analysis are reported in Supplementary Table S2.
