# Supplementary material for: Fluoxetine increases plasticity and modulates the proteomic profile in the adult mouse visual cortex
Source: Sci Rep. 2015 Jul 24;5:12517. doi: 10.1038/srep12517 (PMC4513348; doi:10.1038/srep12517)
Supplement: Supplementary Information [file srep12517-s1.doc]

**TITLE PAGE**

**Fluoxetine increases plasticity and modulates the proteomic profile in the adult mouse visual cortex.**

**L. Ruiz-Perera^1^, M. Muniz^1^, G. Vierci^1^, N. Bornia^1^, L. Baroncelli^2^, A. Sale^2^, F.M. Rossi^1*^**

^1^ Laboratorio de Neurociencias “Neuroplasticity Unit”, Facultad de Ciencias, UdelaR, Montevideo, Uruguay.

^2^ Institute of Neuroscience, Consiglio Nazionale delle Ricerche, Pisa, Italy.

^*^ Corresponding author: F.M. Rossi, Laboratorio de Neurociencias “Neuroplasticity Unit”, Facultad de Ciencias, UdelaR, Iguá 4225, esq. Mataojo, 11400, Montevideo, Uruguay. Tel.: +598 2525 8618 ext. 7138; fax: +598 2525 8617. e-mail: fmrossi@fcien.edu.uy

**Running title:** proteomic of fluoxetine-induced visual cortex plasticity

| **Supplementary Table S1. Proteins with higher level in fluoxetine-treated samples.** | | | | | | | |
| --- | --- | --- | --- | --- | --- | --- | --- |
| **Spot Nº** | **Protein accession** | **Full name** | **Mascot score** | **Nº of matched sequences** | **Nº of matched peptides** | **Protein sequence coverage** | **Peptide sequences**  **confirmed by MS/MS** |
| 464 | ARP2_MOUSE | Actin-related protein 2 | 109 | 9 | 9 | 30% | K.HLWDYTFGPEK.L R.GYAFNHSADFETVR.M |
| 646 | PROF2_MOUSE | Profilin-2 | 256 | 11 | 7 | 61% | K.SQGGEPTY*N*VAVGR.A K.DREGFFTNGLTLGAK.K R.DSLYVDGD*C*T*M*DIR.T |
| 497 | PTPA_MOUSE | Serine/threonine-protein phosphatase 2A activator | 90 | 5 | 5 | 16% | K.FPVIQHFK.F R.WIDETPPVDQPSR.F |
| 690 | CALM_MOUSE | Calmodulin | 60 | 1 | 1 | 10% | -.IDQLTEEQIAEFK.E |
| 613 | TCTP_MOUSE | Translationally-controlled tumor protein | 98 | 3 | 3 | 23% | R.DLISHDELFSDIYK.I |
| 476 | NECP1_MOUSE | Adaptin ear-binding coat-associated protein 1 | 173 | 7 | 7 | 40% | K.LDQPDWTGR.L R.SAFIGIGFTDR.G R.GDAFDFNVSLQDHFK.W |
| 616 | SODM_MOUSE (SOD2) | Superoxide dismutase [Mn], mitochondrial | 133 | 5 | 5 | 31% | K.HHAAYVNNLNATEEK.Y K.AIWNVINWENVTER.Y |
| 605 | PSA2_MOUSE | Proteasome subunit alpha type-2 | 175 | 6 | 6 | 41% | R.GYSFSLTTFSPSGK.L K.HIGLVYSG*M*GPDYR.V K.LAQQYYLVYQEPIPTAQLVQR.V |
| 666 | DYL2_MOUSE | Dynein light chain 2, cytoplasmic | 116 | 3 | 3 | 49% | R.NFGSYVTHETK.H K.YNPTWH*C*IVGR.N |
| 309 | PDIA3_MOUSE | Protein disulfide-isomerase A3 | 186 | 14 | 13 | 30% | K.DASVVGFFR.D K.GFPTIYFSPANK.K R.ELNDFISYLQR.E K.ALEQFLQEYFDGNLKR.Y K.TFSHELSDFGLESTTGEVPVVAIR.T |
| 581 | DHPR_MOUSE | Dihydropteridine reductase | 99 | 9 | 9 | 60% | K.VDAIL*C*VAGGWAGGNAK.S K.RPNSGSLIQVVTTDGK.T |
| 619 | CDC42_MOUSE | Cell division control protein 42 homolog isoform 2 | 195 | 5 | 5 | 33% | K.YVE*C*SALTQR.G K.WVPEITHH*C*PK.T K.TPFLLVGTQIDLR.D |
| 559 | VDAC1_MOUSE | Voltage-dependent anion-selective channel protein 1 | 225 | 8 | 8 | 46% | R.WTEYGLTFTEK.W R.EHINLG*C*DVDFDIAGPSIR.G |
| 544 | ESTD_MOUSE | S-formylglutathione hydrolase | 246 | 7 | 7 | 40% | R.FAVYLPPQAESGK.C K.SGYQQAASEHGLVVIAPDTSPR.G |
| 573 | 1433Z_MOUSE | 14-3-3 protein zeta/delta | 381 | 10 | 9 | 51% | K.DSTLIMQLLR.D K.DSTLI*M*QLLR.D K.GIVDQSQQAYQEAFEISK.K K.TAFDEAIAELDTLSEESYK.D |
| 436 | GLNA_MOUSE | Glutamine synthetase | 348 | 13 | 12 | 33% | R.DIVEAHYR.A R.V*C*EDFGVIATFDPK.P R.LTGFHETSNINDFSAGVANR.G |

**Supplementary Table S1:** The table indicates details of mass spectrometry analysis for the 16 proteins successfully identified which were upregulated by the treatment (see Table 1). The spot number, protein accession number, full name, Mascot score, number of matched sequences, number of matched peptides, the protein sequence coverage and the list of peptide sequences confirmed by MS/MS are reported. Postranslationally modified residues are underlined and in italic (C, cysteine carbamidomethylation; M, methionine oxidation; N, asparagine deamidation).

| **Supplementary Table S2. Proteins with lower level in fluoxetine-treated samples.** | | | | | | | |
| --- | --- | --- | --- | --- | --- | --- | --- |
| **Spot Nº** | **Protein accession** | **Full name** | **Mascot score** | **Nº of matched sequences** | **Nº of matched peptides** | **Protein sequence coverage** | **Peptide sequences**  **confirmed by MS/MS** |
| 328 | SERA_MOUSE | D-3-phosphoglycerate dehydrogenase | 268 | 10 | 10 | 26% | R.GGIVDEGALLR.A K.NAGT*C*LSPAVIVGLLR.E K.EELIAELQD*C*EGLIVR.S |
| 408 | ENOA_MOUSE | Alpha-enolase | 491 | 20 | 17 | 59% | R.IGAEVYHNLK.N R.YITPDQLADLYK.S K.LAQSNGWGV*M*VSHR.S R.AAVPSGASTGIYEALELR.D |
| 481 | ALDOC_MOUSE | Fructose-bisphosphate aldolase C | 118 | 10 | 10 | 37% | R.TPSALAILENANVLAR.Y K.YSPEEIA*M*ATVTALR.R K.GVVPLAGTDGETTTQGLDGLLER.C |
| 524 | MDHC_MOUSE | Malate dehydrogenase, cytoplasmic | 254 | 10 | 9 | 36% | K.ENFS*C*LTR.L K.GEFITTVQQR.G K.FVEGLPINDFSR.E |
| 631 | SYUA_MOUSE | Alpha-synuclein | 401 | 5 | 5 | 42% | K.EGVVHGVTTVAEK.T K.TVEGAGNIAAATGFVK.K K.EQVTNVGGAVVTGVTAVAQK.T |
| 636 | SODC_MOUSE (SOD1) | Superoxide dismutase [Cu-Zn] | 112 | 3 | 3 | 24% | K.KHGGPADEER.H R.VISLSGEHSIIGR.T K.GDGPVQGTIHFEQK.A |
| 639 | NDKA_MOUSE | Nucleoside diphosphate kinase A | 172 | 9 | 8 | 67% | K.DRPFFTGLVK.Y R.TFIAIKPDGVQR.G |
| 689 | HINT1_MOUSE | Histidine triad nucleotide-binding protein 1 | 98 | 4 | 4 | 51% | K.IIFEDDR.C K.AQVAQPGGDTIFGK.I R.*C*LAFHDISPQAPTHFLVIPK.K |

**Supplementary Table S2:** The table indicates details of mass spectrometry analysis for the 8 proteins successfully identified which were downregulated by the treatment (see Table 2). Same abbreviations as in Supplementary Table S1.


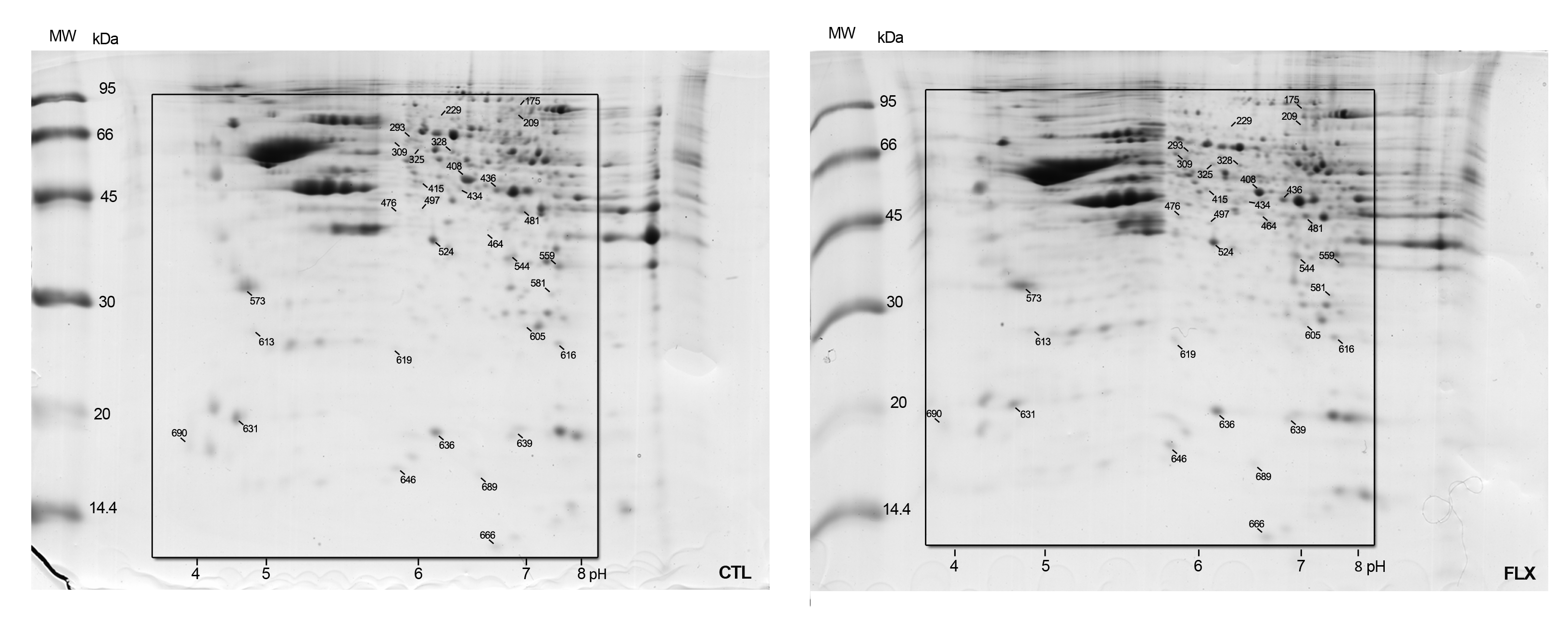


**Supplementary Figure F1:** Fluoxetine modulates the proteomic profile in the adult mouse visual cortex. The figure reports full-length uncropped images of the same 2D gels as in Figure 2. Cropping lines are indicated by a black square. MW, molecular weight (kDa), and pH are indicated. Gels are representative of the 2D proteomic profile of the visual cortex of fluoxetine-treated (FLX) and age-matched (CTL) adult mice, and were stained with colloidal Coomassie Brilliant Blue G250. The 31 spots accepted as significantly differentially expressed between the two experimental conditions are indicated by a line and the corresponding match IDs (see Table 1).


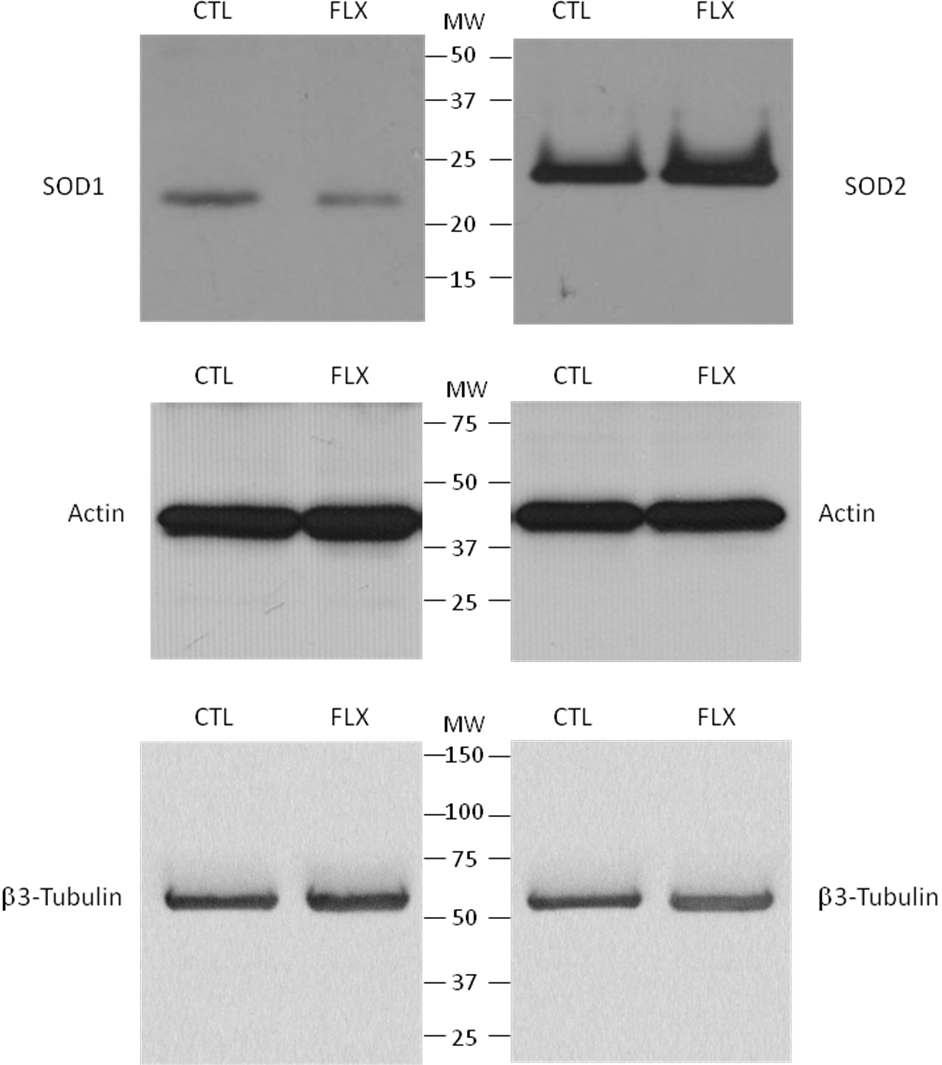


**Supplementary Figure F2:** Validation of 2D gels protein level differences by western blot analysis. Representative western blot of visual cortical samples from fluoxetine-treated (FLX) and age-matched controls (CTL) incubated with anti-SOD1, anti-SOD2, anti-actin or anti-β3-tubulin antibodies. Antibodies recognized a main band at 20-22kDa (SOD1), 25kDa (SOD2), 42 kDa (actin) or 55 kDa (β3-tubulin). Figure reports larger images of the same blots as in Figure 4A. MW, molecular weight (kDa).

| **Supplementary Table S3. Details of fluoxetine treatment.** | | | | | | | |
| --- | --- | --- | --- | --- | --- | --- | --- |
| **cage** | **N° of mice**  **per cage** | **ml consumed per cage** | **ml consumed per mouse** | **mg flx consumed per mouse** | **treatment duration**  **(days)** | **average weight per mouse (g)** | **flx dose per mouse (mg/kg/day)** |
| A | 6 | 752 | 125 | 12.5 | 28 | 28.1±0.7 | 15.9 |
| B | 6 | 789 | 132 | 13.2 | 28 | 27.9±0.6 | 16.8 |
| C | 6 | 784 | 131 | 13.1 | 28 | 28±0.7 | 16.7 |
| D | 6 | 725 | 121 | 12.1 | 28 | 27.8±0.6 | 15.5 |
| E | 6 | 808 | 135 | 13.5 | 28 | 28.6±0.5 | 16.8 |
| F | 6 | 833 | 139 | 13.9 | 28 | 28.9±0.9 | 17.2 |
| **mean** |  | 781.8±17.3 | 130.3±2.9 | 13±0.3 |  | 28.2±0.2 | **16.5±0.3** |

**Supplementary Table S3:** Fluoxetine treatment was performed following drug concentrations and schedules previously used (ref.57, Begenisic et al., 2014). Adult mice were exposed for 4 weeks, from postnatal day (P) 70, to oral fluoxetine (Fluoxetine-hydrochloride, Galeno, Italy or Selectchemie, Laboratorios Gador S.A., Uruguay). Fluoxetine was dissolved in the drinking water in each bottle (at 0.1 mg/ml) for each cage where 6 mice/cage were housed. When fluoxetine solution is substituted with fresh one (twice a week), the volume ingested by the mice in each cage is calculated from the water volume remaining in the bottle. At the end of the treatment with this information and the average weight of the mice housed in each cage, the average amount of fluoxetine ingested per mouse is estimated (approx. 16.5± 0.3 mg/Kg/day/mouse). The table reports details of the fluoxetine (flx) treatment, performed on 6 cages (A-F), each housing 6 mice/cage. The volume (ml) of fluoxetine solution ingested per cage, per mouse, the amount of flx (mg) per mouse, the duration of the treatment (days), the average weight per mouse (g) and the estimated flx dose per mouse (mg/Kg/day) in each cage and the estimated average are reported.
